# A Taguchi-Based and Data-Driven Assessment of Surface Roughness and Wettability in FDM-Printed Polymers

**DOI:** 10.3390/mi17030322

**Published:** 2026-03-05

**Authors:** Mehmet Albaşkara, Eyyup Gerçekcioğlu

**Affiliations:** 1 İscehisar Vocational School, Afyon Kocatepe University, 03200 Afyonkarahisar, Türkiye; 2 Mechanical Engineering Department, Faculty of Engineering, Erciyes University, 38038 Kayseri, Türkiye

**Keywords:** Fused Deposition Modeling (FDM), surface roughness, wettability, Taguchi, ANOVA, artificial neural network (ANN)

## Abstract

Fused Deposition Modeling (FDM) enables rapid, flexible production of polymer-based parts; however, because of additive manufacturing’s nature, it creates distinct microscale surface structures. These micro-scale surface morphologies directly affect the functional properties of the parts, such as surface roughness and wettability. In this study, the surface roughness and contact angle behavior of PLA, PETG, and ABS samples printed via FDM were investigated by varying layer thickness, print orientation, and infill density. The experimental design was created using a Taguchi L16 orthogonal array. Surface roughness was determined by optical profilometry, and wettability was measured by static contact angle tests. Surface topography was supported by scanning electron microscopy (SEM) and three-dimensional surface analyses. The findings revealed that surface roughness is predominantly dependent on layer thickness, whereas wettability is more strongly influenced by printing orientation, which determines the surface’s anisotropy. The developed artificial neural network (ANN) models successfully predicted the trends in surface roughness and contact angle outputs. This study reveals the effect of micro-scale surface structures formed in the FDM process on functional surface behavior, offering a fundamental framework for developing designable surfaces for micromechanical, microfluidic, and biomedical applications.

## 1. Introduction

Additive manufacturing technologies, particularly Fused Deposition Modeling (FDM), have garnered significant interest in both industry and academia in recent years due to their cost-effective production capabilities, geometric flexibility, and ability to work with a wide range of thermoplastic materials [1,2,3,4]. In addition to mechanical performance, the surface properties of parts produced using the FDM method are also critically important for their applications. Surface roughness and wettability parameters significantly affect the final performance of parts across various applications, including biomedical, microfluidic, structural, and in the automotive and aerospace industries [5,6,7].

Surface roughness is a material property strongly influenced by printing parameters, due to the nature of the FDM process. Parameters such as layer height, printing orientation, printing speed, and nozzle temperature lead to surface undulations and distinct layer marks, which are decisive both aesthetically and functionally [8,9,10]. Surface roughness also directly affects the interaction of the surface with liquids, i.e., its wettability. According to the Wenzel and Cassie-Baxter models, the micro- and nanoscale roughness of a surface significantly alters the diffusion behavior of liquid droplets, thereby altering wettability [11]. In addition to the overall roughness level, the size scale and complexity of surface topographic features play a crucial role in determining wettability, as multi-scale and hierarchical structures can significantly alter liquid–surface interactions [12].

Wettability is a fundamental parameter that defines the interaction between a surface and a liquid, thereby determining the surface’s hydrophilic or hydrophobic character. Hydrophilic surfaces are characterized by a low contact angle, while hydrophobic surfaces have a high contact angle. This property is critical not only for liquid retention but also for adhesion, coating, biocompatibility, tribological performance, and cleanability [13,14,15,16]. In particular, wettability directly determines functionality in applications such as facilitating cell adhesion in biomedical implants, ensuring flow control in microfluidic systems, or developing self-cleaning surfaces [4,17,18].

The wettability properties of parts produced by FDM are affected not only by surface roughness but also by the chemical structure of the material used. Poly(lactic acid) (PLA) generally exhibits semi-hydrophilic properties, while polyethylene terephthalate glycol (PETG) tends to be hydrophobic; acrylonitrile butadiene styrene (ABS) can exhibit different behavior depending on its surface energy and morphology [19,20,21]. Therefore, the joint evaluation of material selection and production parameters is crucial for surface engineering.

The relationship between surface roughness and wettability has been studied in the literature. Rough surfaces can increase the contact area between the liquid and the surface, facilitating its spreading on hydrophilic surfaces. Conversely, they can cause the liquid to recede from the surface on hydrophobic surfaces [22,23,24]. This bidirectional effect can create either an advantage or a disadvantage, depending on the application area. Therefore, systematically investigating the relationships between surface roughness and wettability of common thermoplastics, such as PLA, PETG, and ABS, produced by the FDM process, is an important research topic.

Numerous studies in the literature address the surface roughness and wettability properties of parts printed using the FDM method [25,26,27,28,29]. However, studies that systematically examine these two parameters and their microstructural causes for different polymer types within the same experimental framework are limited. In this context, the surface roughness and wettability of PLA, PETG, and ABS samples produced by FDM were investigated across different layer thicknesses, printing angles, and infill densities. The surfaces were examined using scanning electron microscopy (SEM) analysis, and the parameter–output relationship was investigated in detail. In digital manufacturing processes, such as 3D FDM, predicting outputs using machine learning methods, such as artificial neural networks (ANNs), is of great importance. Supporting the experimental results with artificial neural network-based predictive models adds an advanced dimension to the study, not only descriptively but also in predicting micro-scale surface functions. Thus, the aim is to understand the effects of material type and process parameters on surface properties and to develop optimization suggestions for applications.

## 2. Experimental

### 2.1. Materials and Design

**Figure 1:**
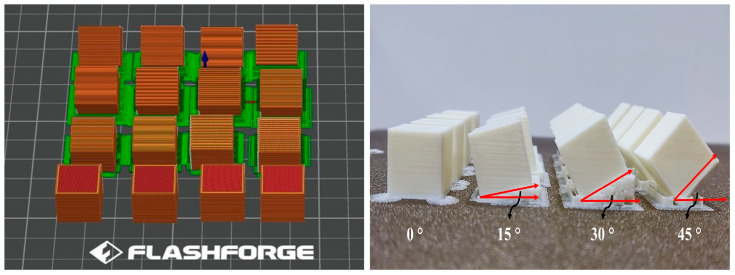
Sample models and printed samples.

In this study, Beta brand 1.75 mm diameter PLA, PETG, and ABS materials, commonly used in the FDM method, were preferred. The fixed printing parameters and filament properties used in the experimental study are given in Table 1. Printing studies were conducted for each filament type at four different layer heights, printing orientations, and infill densities, resulting in 16 different samples printed according to the L16 orthogonal array. For each filament material and each parameter combination, three 15 × 15 mm samples were produced using a Flashforge Adventurer 5M Pro printer (San Po Kong, Hong Kong) according to the experimental design in Table 2. Layer height, printing direction, and infill density were investigated because they critically influence surface formation mechanisms in FDM, including the stepping effect, interlayer adhesion, and subsurface material support, which together determine the final surface properties. The investigated ranges of layer height (0.12–0.21 mm) and infill density (20–50%) were intentionally selected to represent practical and stable FDM printing conditions, as extreme values are known to introduce print instability and excessive surface defects. The models of the samples in the OrcaSlicerslicing software and their post-printing images are presented in Figure 1.

### 2.2. Surface Tests

Surface roughness measurements of the produced samples were performed using a Nanovea (California, USA) S400 optical profilometer. Since the optical profilometer method operates on a non-contact principle, it enables high-precision measurements without damaging the sample surface. The scanning process was performed in a single direction, with a scanning distance of 1 µm and dimensions of 0.4 × 0.4 mm. The obtained data were analyzed using Nanovea Professional 3D software, which applied a Gaussian filter with a cutoff value of 0.05 mm to separate the roughness and ripple components. Three-dimensional surface topography was measured, and the Sa (arithmetic mean height) value was used as the surface roughness parameter. The Sa value provides more accurate measurements in applications where layer traces affect surface quality, such as the FDM method [30]. The Sa parameter was chosen because it provides a representative three-dimensional average roughness value suitable for the periodic layer-induced topographies characteristic of FDM surfaces, and allows for consistent comparison between different materials and process conditions. At least three measurements were taken from different regions for each sample, and average values were calculated. In this way, the effects of printing parameters on surface topography were reliably analyzed.

The surface wettability properties of the samples were determined using the contact angle method on the Attension Theta Lite (Vastra Gotaland, Sweden) device. Distilled water was used in the measurements, and approximately 5 µL of droplets were placed on the surface of each sample via a micropipette. The contact angle of the droplet with the surface was imaged with a high-resolution camera and calculated using software. Three measurements were taken for each sample, and the average contact angle value was calculated. Surfaces with a contact angle less than 90° are considered hydrophilic, while those with an angle greater than 90° are considered hydrophobic.

Scanning electron microscopy (SEM) was performed using a Zeiss Gemini LEO 1430 VP (Oberkochen, Germany) to examine the surface morphology of FDM-printed samples. SEM analyses were used in conjunction with 3D surface profiles to visualize the hill–valley structures specific to the layer-by-layer printing process.

### 2.3. Artificial Neural Network (ANN) Modeling

Artificial neural networks (ANN) were investigated to predict surface roughness (SR) and contact angle (CA) outputs. Artificial neural networks (ANNs) were chosen because of their strong ability to capture the complex, nonlinear interactions frequently observed in additive manufacturing processes. Each material type was added to the ANN model as a parameter. Thus, a total of 48 different experimental models were tested for four parameters and three levels of each parameter. Min–max normalization was applied to all data to ensure the stability of the network during the training process and to mitigate the adverse effects of variable scales on learning. To prevent data leakage, normalization parameters were calculated only from the training subset in each cross-validation step and then applied to the test set. A feedforward regression model was created in MATLAB 2025b to predict SR and CA outputs. The Bayesian Regularization (trainbr) algorithm, which has a high ability to prevent overfitting in small datasets, was preferred [31,32]. This study focuses on evaluating the predictive capability of the Bayesian approach rather than comparing it with alternative machine learning models. Tansig activation functions were used in the hidden layers, and purelin activation functions were used in the output layer. Based on 5-fold cross-validation results, the optimal architectures yielding the highest determination coefficient (R^2^) and lowest mean absolute error (MAE), mean absolute percentage error (MAPE), and root mean square error (RMSE) values were identified.

## 3. Results

### 3.1. Analysis of Results for PLA Samples

Table 3 shows the surface roughness (SR) and contact angle (CA) values obtained with the printing parameters. Although there is no direct linear relationship between the values, some trends are observed. The results show that layer height has the most significant effect on surface roughness. As layer height increases, the layer traces on the surface become more pronounced, and the SR values also increase. In contrast, the CA values do not show a similar pattern. In some experiments, high CA was observed alongside high SR values, whereas in others, high SR was associated with low CA. This indicates that the relationship between surface roughness and wettability is not linear. Due to the nature of 3D printing, the layer traces formed on the surface significantly affect droplet dispersion. Therefore, sometimes low-layer thicknesses resulted in high CA. When each layer thickness is examined separately, it is seen that as roughness increases, CA generally decreases, thus making the surfaces more hydrophilic.

Upon examining the printing orientation, it is observed that the SR values are relatively lower at 0° and 15°, whereas they are higher at 30° and 45°. This shows that the printing orientation affects the surface topography. As the printing angle increases, the stair-step effect becomes more pronounced, resulting in a greater SR [33]. The highest CA values were observed in prints with a printing angle of 0°. As the printing angle increased from 0° to 30°, CA tended to decrease. At 45°, there was generally a slight increase. Infill density does not have a dominant effect on SR and CA.

**Figure 2:**
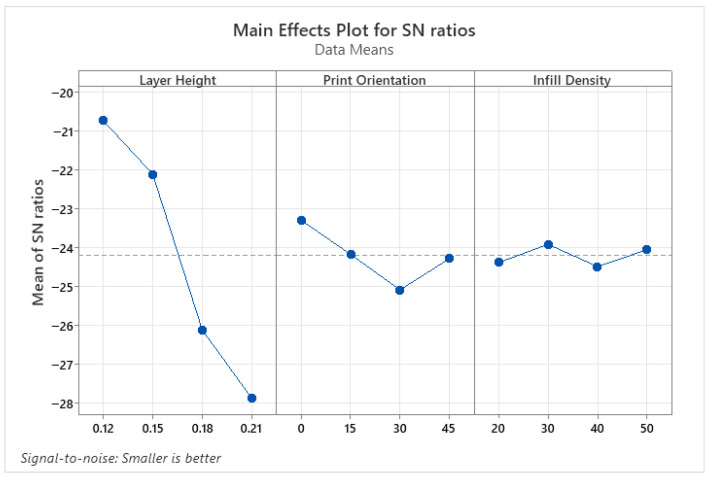
S/N ratio plots for SR of PLA parts.

The parameters affecting surface roughness were identified using the ANOVA results presented in Table 4, and the optimum parameter levels were determined using the S/N ratio tables shown in Figure 2. According to the variance analysis results of the PLA parts, the layer height parameter is an effective parameter for SR, as indicated by *p* < 0.05. This is due to the stair-step effect created by layer-by-layer printing, which is inherent in 3D printing processes. Print orientation and infill density parameters were not found to be effective in improving the quality of the printed object. The optimum parameters for SR were found to be 0.12 for layer height, 0° for print orientation, and 30% for infill density. In conclusion, layer height is the strongest determinant of surface roughness, and print orientation amplifies this effect.

According to the ANOVA results given in Table 5, the only parameter that affects CA is print orientation. Layer height and infill density were not found to be effective for the CA. Print orientation changes the angle at which the layer traces meet the surface [33,34]. This affects the droplet’s adhesion to the surface. Therefore, print orientation has been the most effective parameter. When the CA results in Table 3 are examined, similar to the ANOVA results, they are most affected by print orientation. Thus, it has been determined that contact angle optimization in PLA samples can be achieved primarily by adjusting the print orientation.

**Figure 3:**
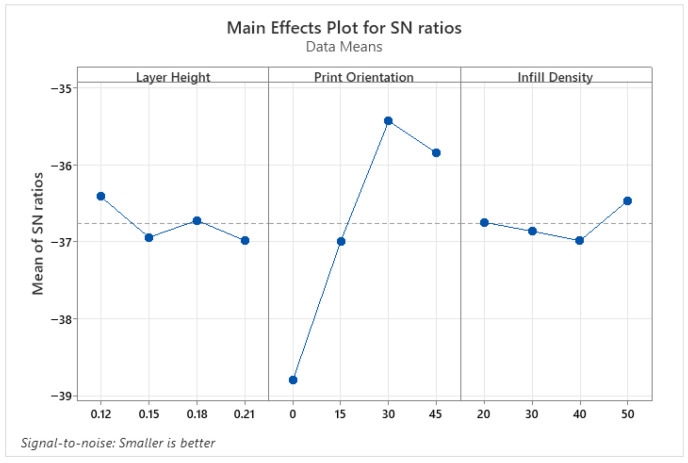
S/N ratio plots for hydrophilic properties of PLA parts.

**Figure 4:**
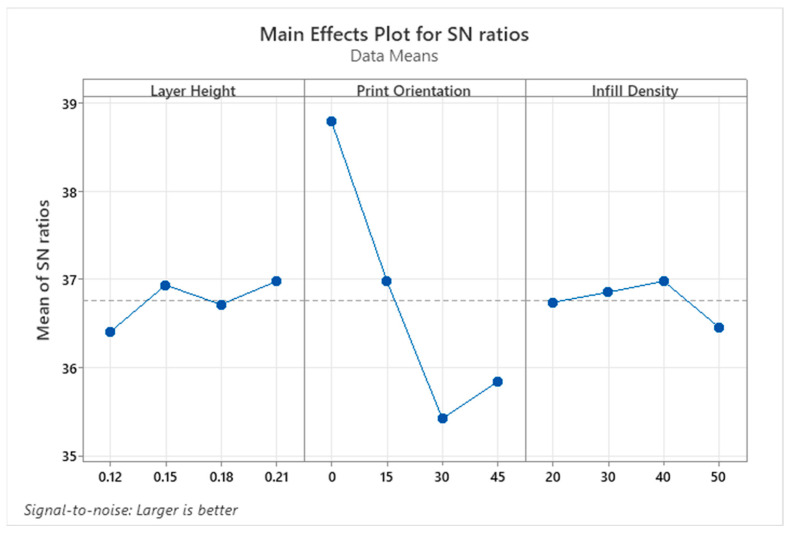
S/N ratio plots for hydrophobic properties of PLA parts.

Wettability results determine whether material surfaces are hydrophilic or hydrophobic. Values with a contact angle greater than 90° are defined as hydrophobic, while values smaller than 90° are defined as hydrophilic. Hydrophilic surfaces are preferred in biomedical, coating, and micro-channel applications, whereas hydrophobic surfaces are preferred in self-cleaning surfaces, water-resistant applications, or those where friction is undesirable [35,36]. Therefore, when determining the optimum parameter levels for CA, levels that provide both hydrophilic and hydrophobic properties were determined. Figure 3 shows the parameter levels that make CA hydrophilic, and Figure 4 shows the parameter levels that make it hydrophobic. Accordingly, the parameter levels that make it hydrophilic are a 0.12 mm layer height, a 30° print orientation, and a 50% infill density, respectively (Figure 3). The levels that make it hydrophobic were found to be 0.21 mm layer height, 0° print orientation, and 40% infill density (Figure 4).

### 3.2. Analysis of Results for PETG Samples

Table 6 presents a comparative analysis of the surface roughness (SR) and contact angle (CA) values of PETG materials. The results indicate that the relationship between SR and CA is not linear, as observed in PLA samples; however, it should be evaluated based on surface topography and material properties. SR values ranged from 9.6 to 24.4 µm, while CA values ranged from 42.9° to 89.9°. In some parameters, contact angle values increased with increasing surface roughness. These results demonstrate that a certain level of roughness prevents droplet adhesion to the surface by creating air pockets, in accordance with the Cassie–Baxter model, and enhances hydrophobicity [18]. In contrast, in experiments 15 and 16, where the SR value was high, the contact angle decreased to 45.6° and 42.9°, respectively. This situation indicates that, as predicted by the Wenzel model, excessive roughness facilitates the penetration of liquid into surface voids, leading to the surface becoming hydrophilic [37].

**Figure 5:**
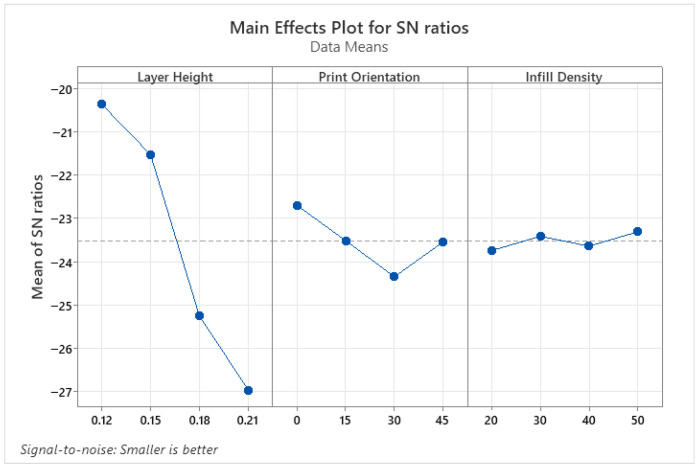
S/N ratio plots for SR of PETG parts.

Table 7 presents the ANOVA results for SR. Accordingly, the most influential parameter on SR is layer height, as in PLA samples. When examining the S/N ratio plots for SR, as shown in Figure 5, the optimum values for SR are 0.12 mm layer height, 0° print orientation, and 50% infill density. These values show that low-layer thickness and horizontally oriented printing parameters have a significant impact on surface roughness.

**Figure 6:**
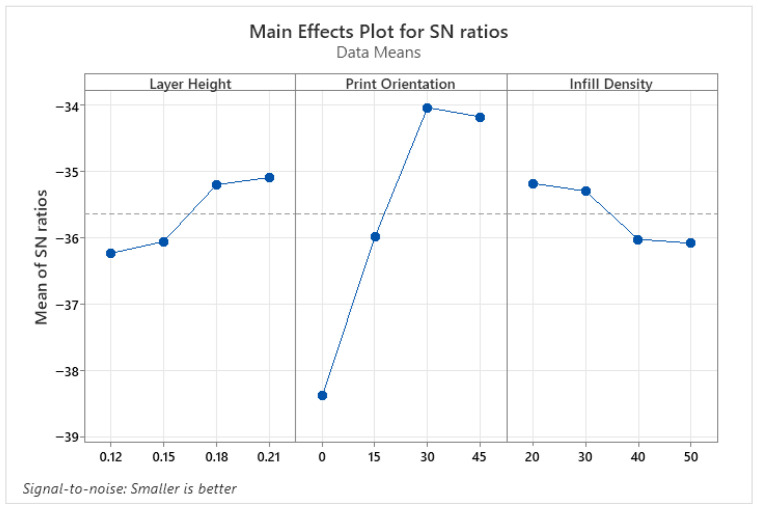
S/N ratio plots for hydrophilic properties of PETG parts.

**Figure 7:**
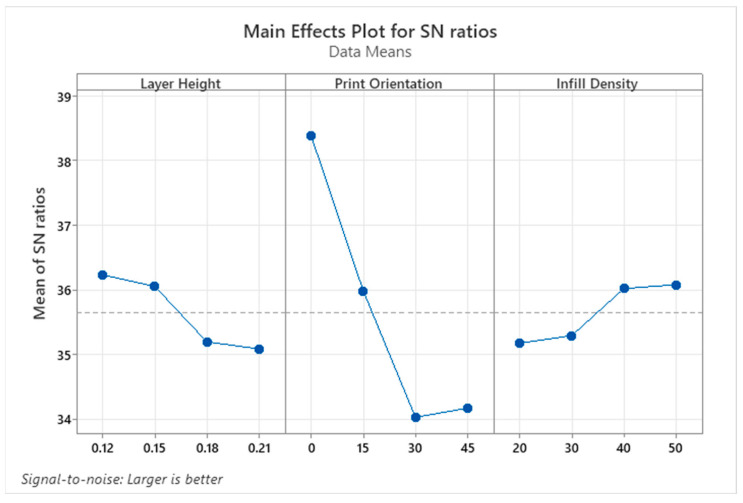
S/N ratio plots for hydrophobic properties of PETG parts.

According to the ANOVA results given in Table 8, the only effective parameter for CA is print orientation. Layer height and infill density do not affect the CA. Figure 6 shows the parameter levels that minimize CA, and Figure 7 shows the parameter levels that maximize CA. A minimum CA indicates that the material is hydrophilic, while a maximum CA indicates that it is hydrophobic. Accordingly, the optimal parameters for making PETG samples hydrophilic are a layer height of 0.21 mm, a 30° print orientation, and a 20% infill density. The optimal parameters for making them hydrophobic are 0.12 mm, 0°, and 50%. Considering that print orientation is the most effective parameter, horizontal print orientation increases hydrophobicity, while a 30° print orientation increases hydrophilicity.

### 3.3. Analysis of Results for ABS Samples

When the surface roughness and contact angle values obtained from ABS samples are examined in Table 9, SR values range from 9.7 to 23.8 µm, while CA values range from 53.8° to 88.3°. The results clearly show that printing parameters significantly affect surface properties in the FDM method. A significant increase in surface roughness values was observed with increasing layer thickness. While SR values were measured in the range of 9.7–13.7 in samples produced with a layer thickness of 0.12 mm, these values increased to the range of 19.1–23.8 at a layer thickness of 0.21 mm. This situation is explained by the stair-stepping effect becoming more pronounced and a coarser topography forming on the surface due to the increase in layer thickness [38]. Printing orientation is also an effective parameter on surface roughness, and SR values increased especially at 30° and 45° orientations due to the prominence of surface markings. The effect of infill density on surface roughness is quite limited compared to layer thickness. Surface roughness values increased slightly at higher infill densities.

Contact angle values showed an inverse trend with surface roughness. Smoother surfaces obtained at lower layer thicknesses yielded lower contact angles. The highest contact angle value of 88.3° was obtained at a layer thickness of 0.21 mm, a printing orientation of 0°, and a coverage of 50%. Increasing the printing orientation significantly affected the contact angle due to the orientation of the surface markings and the formation of an anisotropic surface structure [39].

**Figure 8:**
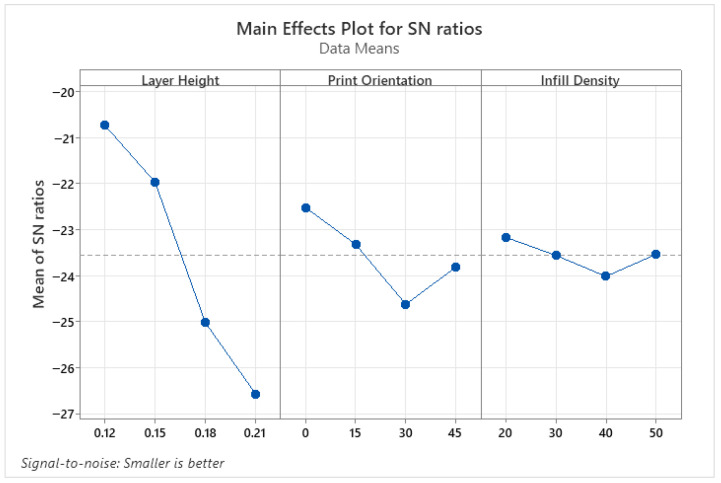
S/N ratio plots for SR of ABS parts.

Table 10, which presents the ANOVA results for SR, shows that the effective parameters are layer height and print orientation. Unlike PLA and PETG samples, print orientation was also found to be an effective parameter for ABS samples. It is thought that the primary reason is the high thermal shrinkage and cooling sensitivity of ABS [40]. Due to its high thermal shrinkage and cooling sensitivity, print orientation has also become effective during the printing of ABS materials. Infill density, on the other hand, is not effective for SR in ABS parts. The S/N ratio plot showing the optimal parameter levels for SR is shown in Figure 8. Accordingly, 0.12 mm layer height, 0° printing angle, and 20% infill density values were determined as effective parameters for the surface roughness of ABS parts. As with PLA and PETG samples, the lowest values of layer height and print orientation were also optimal for ABS.

**Figure 9:**
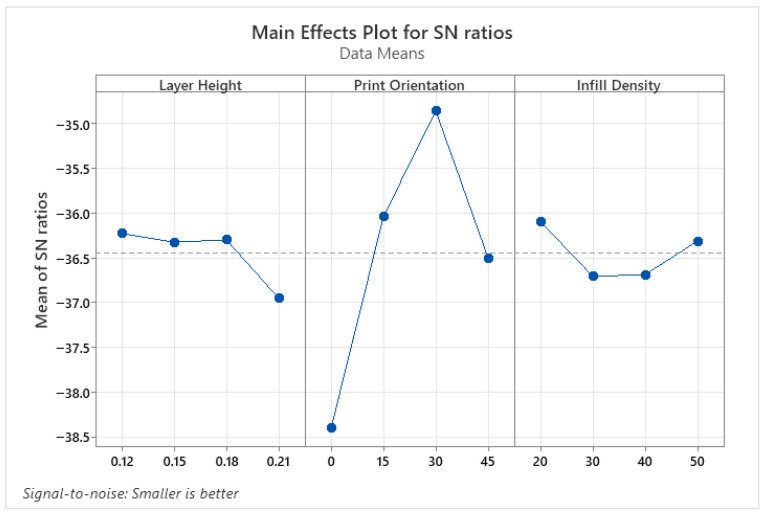
S/N ratio plots for hydrophilic properties of ABS parts.

**Figure 10:**
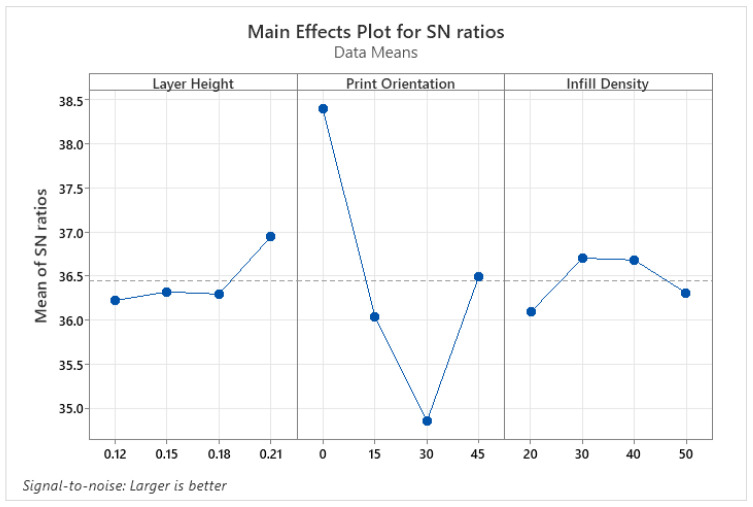
S/N ratio plots for hydrophobic properties of ABS parts.

The ANOVA results obtained for CA are given in Table 11. As with PLA and PETG parts, print orientation was the most effective parameter in ABS samples. The main reason is that print orientation affects the geometric shape of the droplets. To investigate the effects on hydrophilic properties, the S/N plots in Figure 9 showed that the optimum levels were 0.12 mm layer height, 30° print orientation, and 20% infill density. The S/N plot given in Figure 10 shows the optimum levels that make ABS material hydrophobic. Accordingly, a layer height of 0.21 mm, a 0° print orientation, and a 30% infill density are the optimal parameters for making ABS parts hydrophobic. For all material types and print orientations, the most effective CA was 30° for hydrophilic properties and 0° for hydrophobic properties. Thus, it was determined that horizontal printing increases hydrophobicity, while angled printing increases hydrophilicity.

### 3.4. Output Prediction with Machine Learning (ML)

Table 12 shows that the cross-validation (CV) results indicate that the developed ANN models exhibit different but consistent performance for the SR and CA outputs. For the SR output, the RMSE and MAE were 2.14 and 1.63, respectively. The R^2^ value of 0.83 indicates that the model effectively explains a substantial portion of the variance in the data. These results demonstrate that the model has a strong predictive ability for SR and can accurately explain parameter variations and outputs. However, the MAPE of 10.75% for SR is considered a natural consequence of the relatively higher absolute errors arising from the wide dynamic range and high experimental variation in the SR variable.

For the CA output, the RMSE and MAE were 5.9 and 4.4, respectively, and the R^2^ of 0.76 indicated that the model has an acceptable level of explanatory power for CA predictions. The MAPE value of 7.1% for CA indicates that, although the absolute errors are higher than for SR, the relative errors are lower, and the model’s prediction accuracy is at a satisfactory level. This can be attributed to the fact that the CA output has a more limited variance range, and minor absolute deviations have a more significant effect on R^2^. The prediction performance obtained in this study is consistent with previously reported ANN-based models applied to FDM processes. In the literature, ANN models developed for surface roughness prediction typically report R^2^ values in the range of approximately 0.70–0.99, depending on the dataset size, material type, and parameter combinations [41,42,43,44]. The R^2^ values obtained in the present study fall within this commonly reported range, demonstrating that the developed ANN model exhibits a comparable level of predictive ability.

Predictions from the ANN for SR output and the corresponding experimental results are presented in Table 13. Examination of the results reveals that the ANN model attempts to capture surface roughness throughout the entire data distribution. For PLA, the distribution of predictions shows that the model tends to suppress particularly high SR values. This suggests that the ANN cannot fully distinguish the effects of parameters that increase surface roughness in the PLA material and averages them out. In PETG samples, the predicted values exhibit a more uniform distribution around the experimental results. This indicates that ANN exhibits similar behavior under both low and high roughness conditions, providing a better understanding of the surface roughness mechanism. For ABS, the model behavior appears more as local deviations. In some samples, the predictions exceed the experimental value, while in others, they fall below; however, these deviations do not accumulate in a particular direction. Thus, it can be concluded that the ANN model employs a trend-driven learning strategy for SR prediction, indicating that it accurately captures the general increase–decrease behavior of surface roughness. However, it has been found to tend to soften sharp material-specific variations and extreme conditions.

The actual and predicted contact angle values given in Table 14 demonstrate that the ANN model can capture the general wettability trends for PLA, PETG, and ABS. It is noteworthy that the model produces more consistent predictions, particularly in the medium contact angle range, while deviations increase in the low and high contact angle regions. However, the model successfully tracks the general trend of increase and decrease across all material types. Overall, the results demonstrate that despite complex interactions in surface chemistry and microtopography, the ANN approach can predict the contact angle behavior of polymer surfaces produced with FDM with reasonable accuracy, making it a viable method for modeling wettability trends.

### 3.5. Surface Topographies

**Figure 11:**
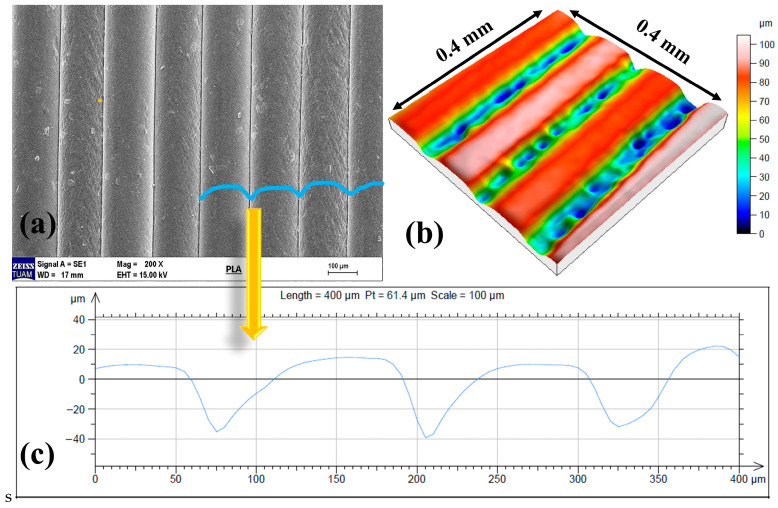
SEM images of the layers (**a**), 3D surface profile (**b**), surface height change (**c**).

The SEM image in Figure 11a clearly shows the distinct and regular layering patterns characteristic of the FDM manufacturing process on the surface of a 3D FDM-printed PLA sample. The linear structures formed by the layer-by-layer deposition of the melt filament demonstrate how the FDM method shapes the surface. This structure indicates that the surface exhibits non-isotropic topography and that roughness varies with the printing direction. The three-dimensional surface topography profile (Figure 11b) further confirms this layered structure observed in the SEM. The successive high and low regions demonstrate the formation of a peak–valley structure along the filament lines. This indicates that the surface roughness is not due to random micro-protrusions, as in many conventional manufacturing methods, but instead to periodic surface undulations caused by the printing geometry. The surface profile analysis in Figure 11c showed that the repetitive height variations observed in the sample printed with a layer height of 0.12 mm were consistent at this layer height. Thus, both ANOVA and SEM images show that the surface structure of samples printed using the 3D FDM method is primarily affected by layer height.

**Figure 12:**
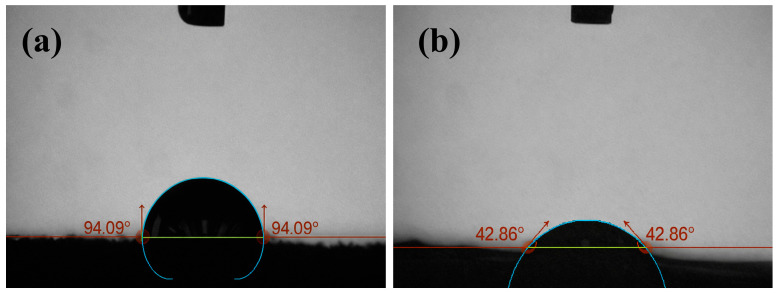
Contact angle measurement images of the highest (**a**), and lowest CA samples (**b**).

Figure 12 shows the highest and lowest contact angle values obtained in the study. The value of 94.09° in Figure 12a represents significant hydrophobic behavior, and the more spherical form of the droplet indicates limited solid–liquid contact. In contrast, the value of 42.86° in Figure 12b shows significant hydrophilic behavior and reveals an increased tendency for the droplet to spread across the surface. The approximately 50° difference in contact angle demonstrates that wettability on FDM surfaces is strongly dependent on both filament type and process parameters. The fact that PLA achieves higher maximum values and PETG shows the lowest minimum value among the three materials confirms that surface topography alone is not the sole determining factor; material-specific surface energy plays a significant role.

### 3.6. Comparison of Materials

When the contact angle values for different materials are examined, it is observed that the filament type has a significant, systematic effect on wettability behavior. In PLA samples, contact angles range from approximately 54.6° to 94.1°, indicating a wide distribution. This indicates that PLA exhibits wettability behavior that is sensitive to changes in printing parameters and the resulting surface roughness. Conditions where particularly high contact angles are observed indicate a more hydrophobic behavior, driven by air trapping and micro-geometric surface features. Low contact angle values suggest greater contact between the liquid and the surface, and that the Wenzel regime may be dominant.

In PETG samples, the contact angle values range from 42.9° to 89.9°, with PETG exhibiting the lowest minimum value among the three materials. This reveals that PETG can show a more hydrophilic character under certain parameter combinations. The wide contact angle range of PETG suggests that both surface topography and material-specific surface energy are effective together. It is understood that, especially under conditions where low contact angles are observed, the liquid penetrates the surface recesses more effectively, and surface chemistry plays a decisive role in wettability.

In ABS samples, contact angle values ranged from 53.8° to 88.3°, generally exhibiting an intermediate behavior between PLA and PETG. The contact angle distribution of ABS shows a hydrophobic tendency in certain parameter combinations, while in others it leans towards a more hydrophilic character. This indicates that ABS surfaces are sensitive to both printing orientation and material chemistry. The observation of different contact angles, even at similar roughness levels, clearly shows that wettability cannot be explained solely by surface roughness.

Overall, the fact that the three filament types exhibit different contact angle ranges despite being produced under similar printing parameters confirms that wettability is multi-factorial. While surface topography plays an important role, material-specific surface energy, chemical structure, and polarity properties also emerge as determining factors. Therefore, it can be concluded that, in the functional surface design of FDM-produced parts, both the printing parameters and the filament selection are critical design variables.

## 4. Conclusions

This study provides a comprehensive evaluation of the effects of filament type and printing parameters on the surface roughness and wettability of FDM printed parts, and the main findings are summarized below:

• The contact angle values of FDM-printed samples varied significantly depending on filament type, ranging from 54.6° to 94.1° for PLA, 42.9° to 89.9° for PETG, and 53.8° to 88.3° for ABS, confirming the strong influence of material-dependent surface chemistry on wettability.
• Even under comparable printing conditions and similar surface morphologies, different polymers exhibited distinct wetting behaviors, demonstrating that wettability is governed by the combined effects of surface topography and intrinsic material surface energy rather than roughness alone.
• PETG exhibited the lowest minimum contact angle (42.9°), indicating the highest hydrophilic tendency under certain parameter combinations, while PLA reached the highest measured contact angle (94.1°), showing the strongest hydrophobic response.
• The variation range of contact angle exceeded 50° across the tested parameter combinations, highlighting the significant tunability of surface wettability through material selection and process parameter control.

Although the ANN model demonstrated successful prediction performance across different material and parameter combinations, its applicability to other materials and extended parameters remains limited due to the inherent dependence of data-driven models on their training dataset. The findings indicate that the surface functionality of polymer parts produced by FDM can be designed in a controlled manner at the microscale through production parameters.

## Figures and Tables

**Table 1:** Fixed printing parameters.

Parameter	Value
Nozzle Temperature (°C)	PLA: 215–PETG: 240–ABS: 260
Table Temperature (°C)	PLA: 60–PETG: 85–ABS: 100
Filament Density (g/cm^3^)	PLA: 1.23–PETG: 1.27–ABS: 1.21
Filament Color	White
Nozzle Diameter (mm)	0.4
Outer Wall Speed (mm/s)	300
Inner Wall Speed (mm/s)	200

**Table 2:** Printing parameters used for each filament.

Exp. No.	Layer Height (mm)	Print Orientation (°)	Infill Density (%)
1	0.12	0	20
2	0.12	15	30
3	0.12	30	40
4	0.12	45	50
5	0.15	0	30
6	0.15	15	20
7	0.15	30	50
8	0.15	45	40
9	0.18	0	40
10	0.18	15	50
11	0.18	30	20
12	0.18	45	30
13	0.21	0	50
14	0.21	15	40
15	0.21	30	30
16	0.21	45	20

**Table 3:** SR and CA results for PLA samples.

Exp. No.	SR (μm)	CA (°)
1	9.6	85.4
2	11.7	64.1
3	12.4	57.6
4	10.1	60.7
5	11.3	89.6
6	11.9	75.4
7	13.7	58.4
8	14.4	61.9
9	19.1	94.1
10	21.2	69.5
11	23.5	54.6
12	17.6	61.8
13	22.1	79.6
14	23.2	74.3
15	26.1	66.3
16	28.1	63.5

**Table 4:** ANOVA for SR of PLA parts.

Source	DF	Adj SS	Adj MS	F-Value	*p*-Value
Layer Height	3	508.085	169.362	42.97	0.0002
Print Orientation	3	23.735	7.912	2.01	0.2145
Infill Density	3	6.430	2.143	0.54	0.6701
Error	6	23.650	3.942		
Total	15	561.900			

**Table 5:** ANOVA for CA of PLA parts.

Source	DF	Adj SS	Adj MS	F-Value	*p*-Value
Layer Height	3	47.01	15.67	0.54	0.674
Print Orientation	3	1904.01	634.67	21.73	0.001
Infill Density	3	50.89	16.96	0.58	0.649
Error	6	175.21	29.20		
Total	15	2177.12			

**Table 6:** SR and CA results for PETG samples.

Exp. No.	SR (μm)	CA (°)
1	9.6	78.5
2	10.9	66.8
3	11.7	56.5
4	9.8	59.5
5	10.9	81.0
6	11.4	67.1
7	12.6	51.8
8	12.8	57.7
9	16.7	82.9
10	18.9	59.3
11	20.9	48.0
12	17.0	46.4
13	19.9	89.9
14	21.3	59.2
15	23.9	45.6
16	24.4	42.9

**Table 7:** ANOVA for SR of PETG samples.

Source	DF	Adj SS	Adj MS	F-Value	*p*-Value
Layer Height	3	371.320	123.773	86.36	0.00003
Print Orientation	3	18.416	6.139	4.28	0.06151
Infill Density	3	3.719	1.240	0.86	0.50884
Error	6	8.599	1.433		
Total	15	402.054			

**Table 8:** ANOVA for CA of PETG samples.

Source	DF	Adj SS	Adj MS	F-Value	*p*-Value
Layer Height	3	126.7	42.24	1.50	0.30662
Print Orientation	3	2743.3	914.42	32.53	0.00042
Infill Density	3	106.1	35.36	1.26	0.36967
Error	6	168.7	28.11		
Total	15	3144.7			

**Table 9:** SR and CA results for ABS samples.

Exp. No.	SR (μm)	CA (°)
1	9.9	82.7
2	9.7	62.8
3	13.7	54.0
4	10.6	62.7
5	11.1	84.4
6	11.7	57.2
7	14.0	53.8
8	13.6	70.8
9	15.1	77.6
10	18.1	61.4
11	18.4	54.5
12	20.0	70.0
13	19.1	88.3
14	22.5	73.2
15	23.8	59.1
16	20.1	64.2

**Table 10:** ANOVA for SR of ABS samples.

Source	DF	Adj SS	Adj MS	F-Value	*p*-Value
Layer Height	3	275.523	91.841	49.02	0.00013
Print Orientation	3	27.462	9.154	4.89	0.04737
Infill Density	3	4.087	1.362	0.73	0.57202
Error	6	11.241	1.874		
Total	15	318.314			

**Table 11:** ANOVA for CA of ABS samples.

Source	DF	Adj SS	Adj MS	F-Value	*p*-Value
Layer Height	3	83.46	27.82	1.29	0.361
Print Orientation	3	1642.67	547.56	25.32	0.001
Infill Density	3	53.18	17.73	0.82	0.529
Error	6	129.75	21.63		
Total	15	1909.07			

**Table 12:** Performance evaluation criteria of ANN.

Output	RMSE	MAE	MAPE (%)	R^2^
SR	2.14	1.63	10.75	0.83
CA	5.9	4.4	7.1	0.76

**Table 13:** Predicted and experimental SR results.

PLA		PETG		ABS	
Actual	Predicted	Actual	Predicted	Actual	Predicted
9.6	10.17	9.6	9.66	9.9	7.65
11.7	11.31	10.9	11.12	9.7	11.95
12.4	12.83	11.7	12.67	13.7	9.54
10.1	12.37	9.8	11.88	10.6	8.90
11.3	12.28	10.9	10.48	11.1	11.59
11.9	15.71	11.4	13.75	11.7	13.94
13.7	13.44	12.6	13.96	14	12.91
14.4	10.49	12.8	12.39	13.6	12.24
19.1	18.60	16.7	16.96	15.1	18.65
21.2	21.62	18.9	19.19	18.1	18.00
23.5	18.19	20.9	17.53	18.4	20.35
17.6	20.78	17	20.19	20	16.88
22.1	24.48	19.9	20.89	19.1	19.83
23.2	23.10	21.3	22.43	22.5	21.96
26.1	23.88	23.9	23.89	23.8	22.86
28.1	23.71	24.4	24.13	20.1	23.17

**Table 14:** Predicted and experimental CA results.

PLA		PETG		ABS	
Actual	Predicted	Actual	Predicted	Actual	Predicted
85.4	86.27	78.5	80.22	82.7	82.85
64.1	72.64	66.8	62.14	62.8	63.75
57.6	58.47	56.5	55.56	54	51.10
60.7	61.68	59.5	57.22	62.7	68.73
89.6	84.57	81	83.55	84.4	81.92
75.4	66.46	67.1	66.56	57.2	64.64
58.4	55.70	51.8	54.43	53.8	56.33
61.9	63.07	57.7	53.96	70.8	64.13
94.1	85.39	82.9	81.75	77.6	89.70
69.5	65.63	59.3	63.03	61.4	65.58
54.6	75.10	48	44.38	54.5	53.81
61.8	63.67	46.4	53.33	70	71.90
79.6	95.84	89.9	78.74	88.3	97.01
74.3	69.25	59.2	72.42	73.2	65.28
66.3	56.69	45.6	58.76	59.1	61.63
63.5	53.98	42.9	45.61	64.2	63.06

## Data Availability

The data presented in this study are available on request from the corresponding authors. Data are not publicly available due to privacy and ethical reasons.
